# Sarcopenia Induced by Chronic Liver Disease in Mice Requires the Expression of the Bile Acids Membrane Receptor TGR5

**DOI:** 10.3390/ijms21217922

**Published:** 2020-10-25

**Authors:** Johanna Abrigo, Fabián Campos, Francisco Gonzalez, Francisco Aguirre, Andrea Gonzalez, Camila Huerta-Salgado, Sabrina Conejeros, Felipe Simon, Marco Arrese, Daniel Cabrera, Alvaro A. Elorza, Claudio Cabello-Verrugio

**Affiliations:** 1 Laboratory of Muscle Pathology, Fragility and Aging, Department of Biological Sciences, Faculty of Life Sciences, Universidad Andres Bello, Santiago 8370146, Chile; j.abrigo.leon@gmail.com (J.A.); fcamposaroca@gmail.com (F.C.); f.gonzalez.wistuba@gmail.com (F.G.); f.aguirregalaz@hotmail.com (F.A.); a.gonzalezrojas@uandresbello.edu (A.G.); c.huertasalgado@uandresbello.edu (C.H.-S.); s.conejeroslillo@uandresbello.edu (S.C.); 2 Millennium Institute on Immunology and Immunotherapy, Santiago 8370146, Chile; fsimon@unab.cl (F.S.); alvaro.elorza@unab.cl (A.A.E.); 3 Center for the Development of Nanoscience and Nanotechnology (CEDENNA), Universidad de Santiago de Chile, Santiago 8350709, Chile; 4 Millennium Nucleus of Ion Channels-Associated Diseases (MiNICAD), Universidad de Chile, Santiago 8370146, Chile; 5 Laboratory of Integrative Physiopathology, Department of Biological Sciences, Faculty of Life Sciences, Universidad Andres Bello, Santiago 8370146, Chile; 6 Centro de Envejecimiento y Regeneración (CARE), Departamento de Gastroenterología, Escuela de Medicina, Facultad de Ciencias Biológicas, Pontificia Universidad Católica de Chile, Santiago 8330077, Chile; marrese@med.puc.cl (M.A.); dacabrer@uc.cl (D.C.); 7 Facultad de Ciencias Médicas, Universidad Bernardo O Higgins, Santiago 8370993, Chile; 8 Faculty of Medicine and Faculty of Life Sciences, Institute of Biomedical Sciences, Universidad Andres Bello, Santiago 8370146, Chile

**Keywords:** bile acids, muscle atrophy, TGR5 receptor, oxidative stress

## Abstract

Sarcopenia is a condition of muscle dysfunction, commonly associated with chronic liver disease (CLD), characterized by a decline in muscle strength, the activation of the ubiquitin-proteasome system (UPS), and oxidative stress. We recently described a murine model of CLD-induced sarcopenia by intake of hepatotoxin 3,5-diethoxycarbonyl-1,4-dihydrocollidine (DDC), which presents an increase in plasma bile acids (BA). BA induced skeletal muscle atrophy through a mechanism dependent on the Takeda G protein-coupled receptor 5 (TGR5) receptor. In the present study, we evaluated the role of TGR5 signaling in the development of sarcopenia using a model of DDC-induced CLD in C57BL6 *wild-type* (WT) mice and mice deficient in TGR5 expression (TGR5^−/−^ mice). The results indicate that the decline in muscle function and contractibility induced by the DDC diet is dependent on TGR5 expression. TGR5 dependence was also observed for the decrease in fiber diameter and sarcomeric proteins, as well as for the fast-to-slow shift in muscle fiber type. UPS overactivation, indicated by increased atrogin-1/MAFbx (atrogin-1) and muscle RING-finger protein-1 (MuRF-1) protein levels and oxidative stress, was abolished in tibialis anterior muscles from TGR5^−/−^ mice. Our results collectively suggest that all sarcopenia features induced by the DDC-supplemented diet in mice are dependent on TGR5 receptor expression.

## 1. Introduction

Sarcopenia is characterized by low skeletal muscle strength, decreased muscle mass, and low physical performance [1]. Sarcopenia can be classified as primary when associated with aging or secondary when related to nutritional disorders, limited mobility, and chronic diseases [2,3]. Sarcopenic skeletal muscle shows a decrease in fiber diameter, fiber transition, and reduced content of sarcomeric proteins, such as myosin heavy chain (MHC) and troponin I [4]. Several alterations are also observed at the molecular level. One of these is the increased activity and expression of the ubiquitin-proteasome system (UPS), specifically the E3 ubiquitin ligases atrogin-1 and MuRF-1. Sarcopenic muscle likewise develops oxidative stress, characterized by the increased production of reactive oxygen species (ROS) and oxidative-dependent protein modifications, such as the formation of 4-hydroxynonenal (4-HNE) adducts in proteins. Other intracellular events altered in atrophic muscles are mitochondrial dysfunction, autophagy, and myonuclear apoptosis [4,5].

Sarcopenia is one of the most critical and prevalent comorbidities related to the development and progression of chronic liver disease (CLD) [6,7,8,9]. Muscle wasting is associated with functional decline, frailty, disability, hospitalization, and increased morbidity and mortality in patients with CLD [8,10,11,12]. Furthermore, sarcopenia is an independent predictor of pre- and post-liver transplantation mortality [13]. Recently, we described a murine model of CLD-associated sarcopenia by intake of hepatotoxin 3,5-diethoxycarbonyl-1,4-dihydrocollidine (DDC). This model presents a decrease in muscle strength and functionality, a fiber type transition, and a reduction in fiber diameter and MHC levels. We also found an increase in ROS levels and atrogin-1 and MuRF-1 protein levels [5,14].

Cholestatic CLD, such as the DDC-induced liver disease model, is associated with a marked increase in the plasma levels of bile acids (BA). Of note, the BA concentration increases almost 1000-fold in DDC-treated animals compared with healthy humans or mice, possibly triggering several side effects in extrahepatic tissues [15]. BA act as signaling molecules and may activate several receptors, such as the nuclear receptor farnesoid X receptor and the membrane Takeda G protein-coupled receptor 5 (TGR5) [16,17]. TGR5 was the first described cell surface receptor-activated BA and is expressed in different cell types, such as hepatic cells (Kupffer cells, cholangiocytes, sinusoidal endothelial cells, and activated hepatic stellate cells), macrophages, and adipocytes. TGR5 is also expressed in other tissues, such as the pancreas, gallbladder, kidney, spleen, intestine, and skeletal muscle [16,18]. The TGR5 receptor is involved in regulating energy homeostasis, glucose metabolism, and inflammatory responses [19].

TGR5 is the unique receptor for BA expressed in skeletal muscle, an area in which its function is not fully understood. Recent evidence suggests that TGR5 and its endogenous ligands could regulate normal skeletal muscle differentiation [20,21,22]. We have recently demonstrated that cholic and deoxycholic acids induce muscle atrophy in healthy muscle fibers via a mechanism dependent on the TGR5 receptor [23].

To date, there is no information on the role of the TGR5 receptor in regulating muscle mass under pathological conditions such as CLD. In this study we aim to determine the participation of TGR5 expression in sarcopenia induced by cholestatic CLD in mice.

## 2. Results

### 2.1. TGR5 Ablation Does Not Alter the Increased Plasma BA in Mice with CLD, but It Protects from the Decline in Muscle Mass

Cholestatic CLD produces DDC-induced sarcopenia. Thus, we performed an analysis of parameters indicative of hepatic injury in *wild-type* (WT) and TGR5^−/−^ mice. Table 1 shows that the DDC-supplemented diet induces an increase in liver damage parameters, such as ALT and ALP activities, total bilirubin levels, and an increase in the liver size of WT and TGR5^−/−^ mice. The results indicate that DDC hepatotoxin induces CLD independent of TGR5 expression in mice for at least 6 weeks because WT and TGR5^−/−^ mice were similarly affected. The glycemic index was also decreased in the DDC-fed mice independently of TGR5 expression. Table 1 shows that the fat amount decreases in WT mice fed with the DDC diet. In TGR5^−/−^ mice, the basal fat amount is increased compared with that of WT mice, and the DDC diet maintains this increase. This result is consistent with the role of TGR5 in adipose tissue [24]. In Table 1, we observed that the hydration ratio was similar in WT and TGR5^−/−^ mice, which indicates that mice in all experimental conditions were hydrated as healthy mice.

We have recently reported that BA induce muscle atrophy in muscle fibers through a mechanism dependent on the TGR5 receptor [23]. Supplementary Figure S1 shows that the DDC diet increases the plasma levels of BA in WT and TGR5^−/−^ mice (WT-chow = 1.0 ± 11; WT-DDC = 1200 ± 100; TGR5^−/−^ chow = 1.5 ± 20; TGR5^−/−^ DDC = 1100 ± 72). Then, we evaluated TGR5 activation in tibialis anterior (TA) muscles from WT and TGR5^−/−^ mice. The measurement of TGR5-dependent transcriptional activity was achieved by the electroporation of the pCRE-luc plasmid reporter. Supplementary Figure S2A shows that the DDC diet induces pCRE-luc activity in WT mice (chow = 1.00 ± 0.37; DDC = 2.67 ± 0.21), whereas this effect is lost in TGR5^−/−^ mice (chow = 0.30 ± 0.05; DDC = 0.21 ± 0.03). These results are in accordance with the data presented in Supplementary Figure S2B, which shows TGR5 expression in TA muscles from WT mice (chow: 3.34 ± 0.16-fold; WT-DDC: 3.51 ± 0.36-fold). TGR5 expression is absent in TGR5^−/−^ mice.

The mass of specific muscles was also evaluated. Table 1 shows that in WT mice there is a DDC-induced decline in the muscle mass of the TA, gastrocnemius (GAST), extensor digitorum longus (EDL), and soleus (SOL). In the same table, we show that the muscles’ weight remains unchanged in TGR5^−/−^ mice independently of the administered diet (i.e., chow or DDC diet).

These results show that CLD induced by the DDC diet increases the plasma BA in WT and TGR5^−/−^ mice; however, it only decreases muscle mass in WT mice. TGR5-dependent signaling increases in the TA muscle from WT mice fed with the DDC diet but not in TGR5^−/−^ mice.

### 2.2. Muscle Function Declines in Mice with CLD via a Mechanism Dependent on TGR5 Expression

**Figure 1:**
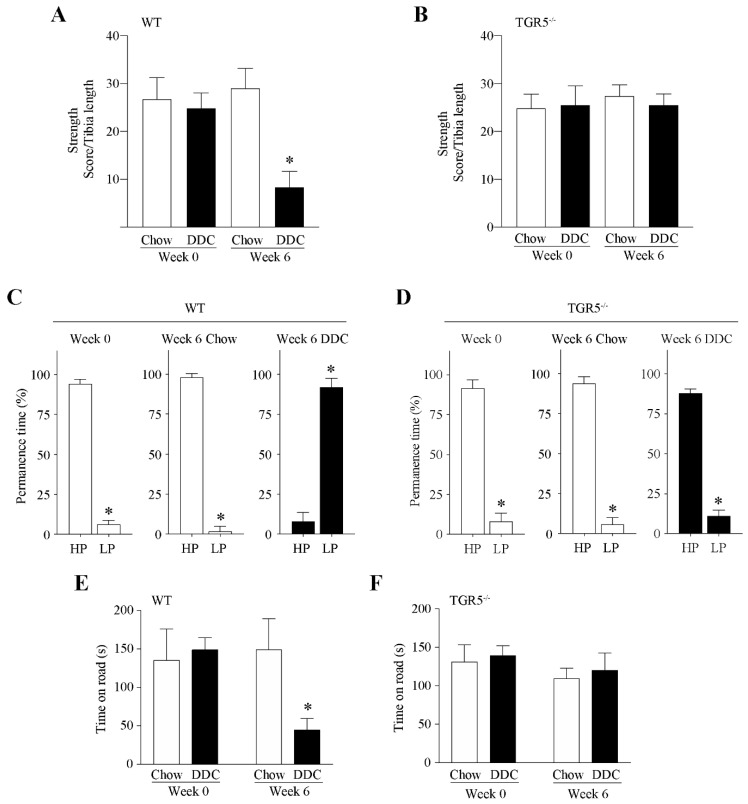
Muscle function declines in mice with CLD via a mechanism dependent on TGR5 expression. C57BL/6J male WT and TGR5^−/−^ mice were fed with a chow or a DDC-supplemented diet for 6 weeks. The weightlifting test strength measure was evaluated in WT (**A**) and TGR5^−/−^ (**B**) mice. The values correspond to the mean ± standard error of the mean (SEM) (*n* = 7–9, * *p* < 0.05 vs. chow at week 0, one-way ANOVA, Tukey’s multiple comparison test). The ability to perform physical exercise was evaluated with a treadmill test in WT (**C**) and TGR5^−/−^ mice (**D**). The running mice were recorded by video, and the permanence time in seconds was calculated and expressed as the percentage of time spent in each zone: high (HP) and low (LP) performance zones of the treadmill (*n* = 7–9, * *p* < 0.05 vs. WT-chow, *t*-test). The rotarod test was performed in WT (**E**) and TGR5^−/−^ (**F**) mice, and the time (s) on the rod was determined. The values correspond to the mean ± SEM (*n* = 7–9, * *p* < 0.05 vs. chow at week 0, two-way ANOVA, Tukey’s multiple comparison test).

Under these conditions, we evaluated muscle function based on a muscle strength test in live mice. For this evaluation, we performed the weightlifting test. Figure 1A shows that WT-DDC mice have less ability to support weight than the WT-chow group at six weeks of treatment (WT-chow = 29.00 ± 4.21 score; WT-DDC = 8.38 ± 3.29). On the contrary, Figure 1B shows that TGR5^−/−^ mice did not present differences between mice fed with chow and mice fed with the DDC diet (TGR5^−/−^ chow = 27.44 ± 2.30; TGR5^−/−^ DDC = 25.56 ± 3.12). Thus, the muscle strength in live mice that express the TGR5 receptor is decreased by DDC-induced CLD.

Then, we evaluated physical activity through a treadmill test. Figure 1C shows that WT mice fed with the DDC diet for six weeks spend more time in the low performance (LP) region and less time in the high performance (HP) zone compared with the WT-chow group (WT-chow: HP = 98.18% ± 2.51% and LP = 1.82% ± 0.43%; WT-DDC: HP = 8.07% ± 5.52% and LP = 91.93% ± 6.31%), which indicates that WT-DDC mice present decreased physical activity. Figure 1D shows that DDC treatment did not affect the performance of TGR5^−/−^ mice (TGR5^−/−^ chow: HP = 94.00% ± 4.11% and LP = 6.00% ± 3.43%; TGR5^−/−^ DDC: HP = 88.13% ± 2.59% and LP = 11.25% ± 3.54%), which is similar to the WT-chow group. Thus, physical activity is decreased by the DDC diet only in mice that express the TGR5 receptor.

To complement the above tests, we conducted the rotarod test. Figure 1E shows that at six weeks of treatment, WT-DDC mice present a decreased time on the rod compared with WT-chow mice (WT-chow = 148.6 ± 18.2 s; WT-DDC = 44.4 ± 6.9 s). Figure 1F shows that at six weeks of treatment, TGR5^−/−^ mice were refractory to the decrease in time spent on the rod (TGR5^−/−^ chow =109.2 ± 13.5 s; TGR5^−/−^ DDC = 120.0 ± 22.4 s).

These results suggest that the decline in muscle function of mice with CLD is dependent upon the TGR5 receptor.

### 2.3. TGR5 Genetic Ablation Protects Mice with CLD from a Decrease in Muscle Strength and an Increase in Fatigue

**Figure 2:**
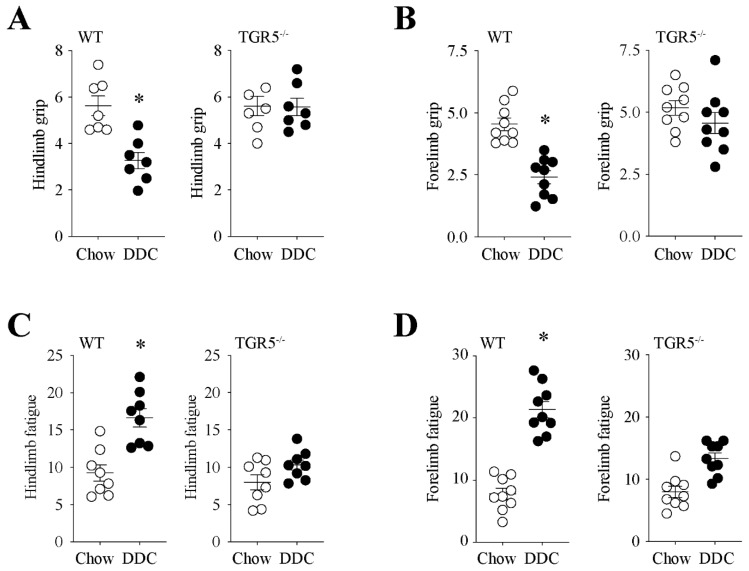
The absence of TGR5 expression in mice abolishes the DDC-diet-induced resistance to fatigue and the decrease in muscle strength. C57BL/6J male WT and TGR5^−/−^ mice were fed with a chow or DDC-supplemented diet for 6 weeks. The handgrip test in the hindlimb (**A**,**C**) and forelimb (**B**,**D**) was performed at the end of the treatments to measure strength (**A**,**B**) and fatigue (**C**,**D**). The values correspond to the mean ± SEM (*n* = 7–9, * *p* < 0.05 vs. chow, *t*-test).

We determined the effect of TGR5 on muscle strength. To measure this, we evaluated the strength and fatigue in living mice using the handgrip test. Our results show that after administration of the DDC diet, handgrips in the hindlimb (Figure 2A) and forelimb (Figure 2B) are decreased in WT mice compared with chow diet mice (hindlimb: WT-chow = 5.62 ± 1.12 vs. WT-DDC = 3.27 ± 0.94; forelimb: WT-chow = 4.55 ± 0.77 vs. WT-DDC = 2.41 ± 0.77). The same figures show that TGR5^−/−^ mice are resistant to the DDC-induced diminution in strength in both the hindlimb and forelimb (hindlimb: TGR5^−/−^-chow = 5.61 ± 1.10 vs. TGR5^−/−^-DDC = 5.57 ± 0.99; forelimb: TGR5^−/−^-chow = 5.18 ± 0.89 vs. TGR5^−/−^-DDC = 4.57 ± 0.25). The handgrip test also allows us to determine fatigue. Figure 2C,D show the evaluation of fatigue in the hindlimb and forelimb, respectively. The results indicate that fatigue increased in WT-DDC mice compared with WT-chow mice in both the hindlimb and forelimb (hindlimb: WT-chow = 9.26 ± 3.11 vs. WT-DDC = 16.65 ± 3.54; forelimb: WT-chow = 7.82 ± 0.77 vs. WT-DDC = 21.37 ± 4.00). Figure 2C,D show that TGR5^−/−^ mice did not present a change in fatigue measurements (hindlimb: TGR5^−/−^-chow = 7.99 ± 2.85 vs. TGR5^−/−^-DDC = 10.33 ± 1.95; forelimb: TGR5^−/−^-chow = 8.91 ± 2.74 vs. TGR5^−/−^-DDC = 13.36 ± 2.57).

**Figure 3:**
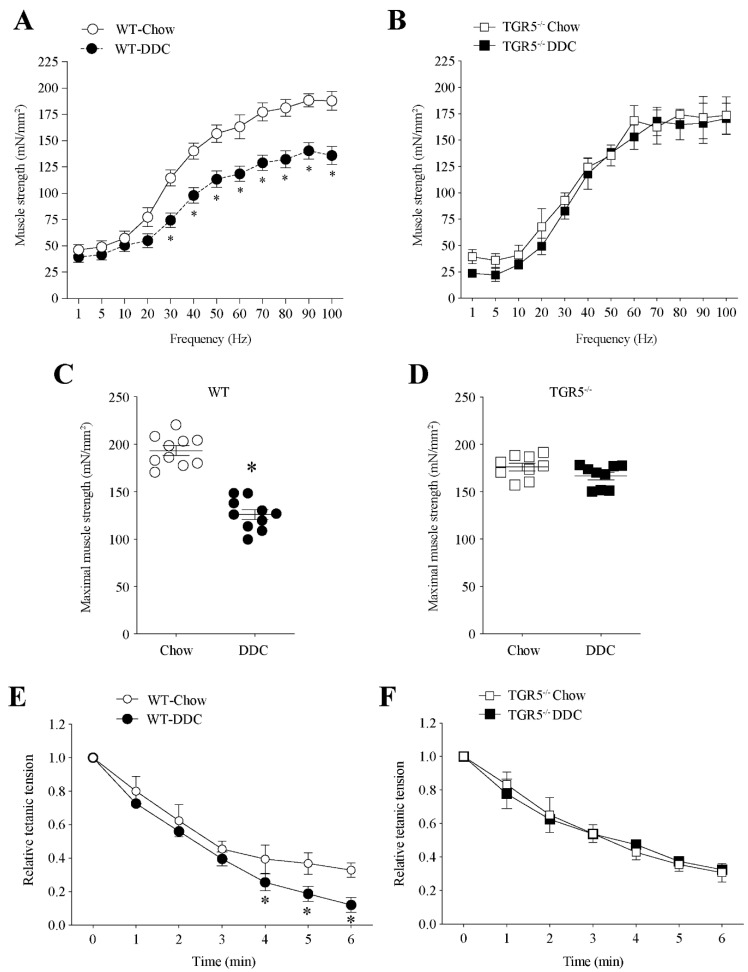
The decreased tetanic force in mice with DDC-induced CLD is dependent on TGR5 expression. C57BL/6J male WT and TGR5^−/−^ mice were fed with a chow or DDC-supplemented diet for 6 weeks. When the experiment was completed, the tibial anterior (TA) muscle was dissected, and a curve of force versus frequency was determined for WT (**A**) and TGR5^−/−^ (**B**) mice. The values correspond to the mean ± SEM (*n* = 7–9, * *p* < 0.05, two-way ANOVA, Sidak’s multiple comparison test). The maximal tetanic force for WT (**C**) and TGR5^−/−^ (**D**) mice was plotted. The values correspond to the mean ± SEM (*n* = 7–9, * *p* < 0.05 vs. chow, *t*-test). Fatigue determination was evaluated in the TA of WT (**E**) and TGR5^−/−^ (**F**) mice. The values correspond to the mean ± SEM (*n* = 7–9, * *p* < 0.05, two-way ANOVA, Sidak’s multiple comparison test).

We likewise evaluated muscle tension in isolated TA muscles through electrophysiological measures. Figure 3A shows that WT-DDC mice present decreased tension compared to WT-chow mice at frequencies higher than 30 Hz. Figure 3B shows that in TGR5^−/−^ mice, the DDC diet did not change the tension generated by TA muscles compared to mice fed with the chow diet. We calculated the tetanic force from the curves of frequency vs. tension, as shown in Figure 3A,B. The data show that the decreased tetanic strength in the TA muscle observed in WT-DDC mice (Figure 3C; WT-chow = 193.3 ± 5.5 vs. WT-DDC = 134.9 ± 5.0) is prevented in TGR5^−/−^-DDC mice (Figure 3D; TGR5^−/−^-chow: 176.4 ± 4.5 vs. TGR5^−/−^-DDC: 166.6 ± 4.8).

Another contractile property of isolated muscle is fatigue in response to electrical stimulation. Figure 3E shows the increased fatigue (between 4 and 6 min of the test) of the TA muscle from WT-DDC compared with WT-chow mice. At the end of the measurement (6 min), we found a 62.9% decrease in tension of WT-DDC mice in the WT-chow group (WT-chow = 0.33 ± 0.03 vs. WT-DDC = 0.12 ± 0.03). Figure 3F shows a similar tension curve comparing TGR5^−/−^ mice fed with the chow or DDC diet (At 6 min: chow = 0.31 ± 0.04 vs. DDC = 0.32 ± 0.02).

Altogether, our data indicate that the decrease in muscle strength and the increase in muscle fatigue induced by CLD are protected against by the absence of TGR5 expression in mice.

### 2.4. CLD-Induced Fast-to-Slow Shift in Muscle Fiber Type Composition Is Dependent on TGR5 Expression

**Figure 4:**
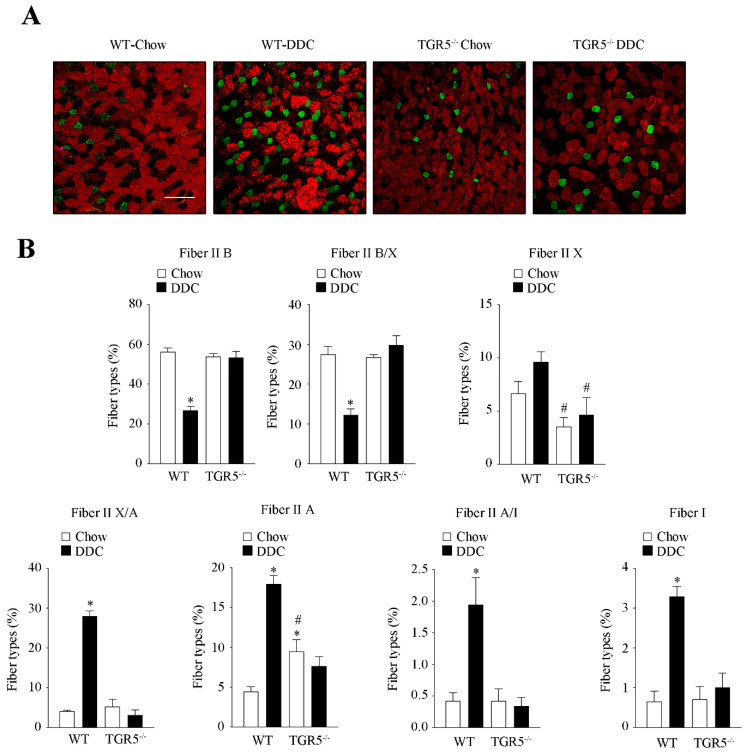
The fast-to-slow transition in muscle fiber type induced by CLD is dependent on TGR5 expression. The TA muscle was obtained from C57BL/6J male WT and TGR5^−/−^ mice fed with a standard (chow) or DDC-supplemented diet for 6 weeks. (**A**) The fiber types were determined by indirect immunofluorescence detection of MHC isoforms (IIA, IIB, I). Fluorescent images reveal the fiber composition in a whole muscle/cross-section: red (IIB), green (IIA), blue (I), not stained (IIX), and mixed: IIB/IIX, IIA/IIX, IIA/I. The scale bar corresponds to 150 µm. (**B**) The abundance of the different fiber types in the experimental groups (WT-chow, WT-DDC, TGR5^−/−^-chow, and TGR5^−/−^-DDC) is shown in the graphs. The values represent the mean ± SEM (*n* = 3, * *p* < 0.05 vs. chow, # *p* < 0.05 vs. WT DDC, two-way ANOVA, Tukey’s multiple comparison test).

We evaluated the effect of TGR5 expression on the transition of muscle fiber types induced by the DDC diet in mice. Figure 4A shows the images for the detection of fiber types from TA muscles in the different experimental conditions. Figure 4B shows that the changes produced by DDC in WT mice (fiber type IIB: chow = 56.20 ± 1.13 vs. DDC = 26.86 ± 1.20; IIB/X: chow = 27.52 ± 2.10 vs. DDC = 12.25 ± 1.51; IIX: chow = 6.66 ± 1.14 vs. DDC = 9.63 ± 0.96; IIX/A: chow = 4.09 ± 0.31 vs. DDC = 28.02 ± 1.23; IIA: chow = 4.45 ± 0.62 vs. DDC = 18.01 ± 1.02; IIA/I: chow = 0.42 ± 0.13 vs. DDC = 1.94 ± 0.43; I: chow = 0.66 ± 0.26 vs. DDC = 3.29 ± 0.24) are prevented by the absence of TGR5 expression (IIB: chow = 53.86 ± 0.93 vs. DDC = 53.37 ± 1.75; IIB/X: chow = 26.75 ± 0.64 vs. DDC = 29.86 ± 2.29; IIX: chow = 3.53 ± 0.85 vs. DDC = 4.68 ± 1.58; IIX/A: chow = 5.28 ± 1.79 vs. DDC = 3.13 ± 1.35; IIA: chow = 9.49 ± 1.50 vs. DDC = 7.68 ± 1.16; IIA/I: chow = 0.42 ± 0.19 vs. DDC = 0.35 ± 0.13; I: chow = 0.71 ± 0.31 vs. DDC = 1.01 ± 0.36). In general, we observed that the glycolytic profile of fibers in TA from WT mice fed with the DDC diet is changed, becoming more oxidative with an increase in the abundance of fiber types I, IIA/I, IIA and IIX/A, whereas there is a decreased proportion of IIB fibers. This is not observed in the TA fibers from TGR5^−/−^ mice fed with the DDC diet.

### 2.5. TGR5 Ablation Protects the Muscle from a Decrease in Fiber Diameter Induced by CLD in Mice

**Figure 5:**
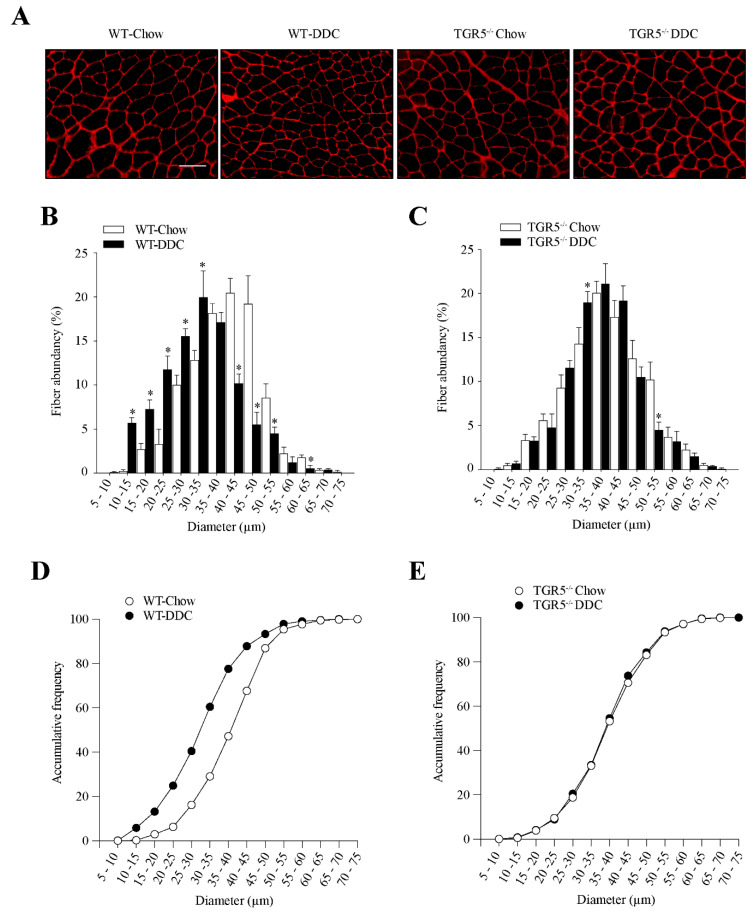
The absence of TGR5 expression in mice abolishes the DDC diet-induced diminution of fiber diameter. C57BL/6J male WT and TGR5^−/−^ mice were fed with a chow or DDC-supplemented diet for 6 weeks. (**A**) TA muscle cross-sections were stained with wheat germ agglutinin (WGA) to delimit muscle fiber sarcolemma. The scale bar corresponds to 100 µm. Minimal Feret’s diameters were determined using ImageJ software. Fiber diameters were grouped from 0 to 75 μm, and the values were expressed as the percentage of the total fibers quantified for WT (**B**) and TGR5^−/−^ mice (**C**). Values correspond to the mean ± SEM (*n* = 3, * *p* < 0.05, two-way ANOVA, Sidak’s multiple comparison test). Accumulative frequency analysis for WT (**D**) and TGR5^−/−^ (**E**) mice were plotted.

We evaluated the effect of TGR5 ablation on fiber diameter after treatment with the DDC diet. Figure 5A shows the detection of fiber diameter by wheat germ agglutinin (WGA) staining in transversal slides of TA muscles. Figure 5B presents the distribution of abundance for fiber diameter in muscles from WT mice. This figure shows that the DDC diet generates a high quantity of smaller fiber sizes than the WT-chow group. Figure 5C shows that the TA muscles from TGR5^−/−^ mice fed with the chow or DDC diets present a similar size distribution for fiber diameter. An analysis of cumulative frequency indicates that in WT mice the DDC diet displaced the high abundance toward smaller size, compared with the chow diet (Figure 5D). Thus, we can observe that between 0 and 40 µm, the cumulative frequency shown by WT mice fed with the DDC diet is higher than that reached by mice fed with the chow diet (chow = 47.2% vs. DDC = 77.6%). On the other hand, the same analysis performed with TGR5^−/−^ mice (Figure 5E) does not show a difference between the chow and DDC diets (0 to 40 µm: chow = 53.2% vs. DDC = 54.6%).

Altogether, the results indicate that the DDC diet decreases the fiber diameter in TA muscles from WT mice, but this effect is lost in TA muscles from TGR5^−/−^ mice.

### 2.6. The Decreased Levels of Sarcomeric Protein and the Increased UPS Activity Induced by CLD Are Dependent on TGR5 Expression

**Figure 6:**
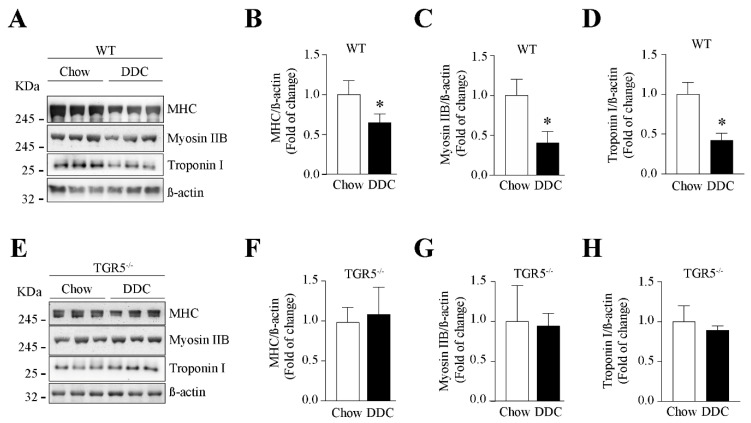
TGR5 participates in reducing the myosin heavy chain (MHC) protein levels in mice with CLD. C57BL/6J male WT and TGR5^−/−^ mice were fed with a chow or DDC-supplemented diet for 6 weeks. When the experiment was completed, TA muscles were excised and homogenized to evaluate MHC protein levels, the IIB isoform of myosin, troponin I in WT (**A**), and TGR5^−/−^ (**E**) mice. β-actin was used as the loading control, and the molecular weight markers are depicted in kilodaltons (kDa). The quantitative analysis for MHC (**B**,**F**), IIB myosin (**C**,**G**), and troponin I (**D**,**H**) protein levels from WT (**B**–**D**) and TGR5^−/−^ (**F**–**H**) mice are shown. The values represent the mean ± SEM (*n* = 6, * *p* < 0.05 vs. chow, *t*-test).

We determined the effect of TGR5 expression on the levels of sarcomeric proteins in TA muscles. Figure 6A,E shows the protein levels of MHC, myosin IIB, and troponin I evaluated in the TA muscle from WT and TGR5^−/−^ mice, respectively. WT-DDC mice show a decrease in MHC (Figure 6B; chow = 1.00 ± 0.05 vs. DDC = 0.65 ± 0.04), myosin IIB (Figure 6C; chow = 1.00 ± 0.12 vs. DDC = 0.40 ± 0.09), and troponin I (Figure 6D; chow = 1.00 ± 0.09 vs. DDC = 0.42 ± 0.05) protein levels. On the contrary, TGR5^−/−^-DDC mice do not show changes in the levels of these proteins (Figure 6F, MHC: chow = 1.00 ± 0.11 vs. DDC = 1.08 ± 0.20; Figure 6G, myosin IIB: chow = 1.00 ± 0.28 vs. DDC = 0.94 ± 0.09; Figure 6H, troponin I: chow = 1.00 ± 0.12 vs. DDC = 0.89 ± 0.04).

**Figure 7:**
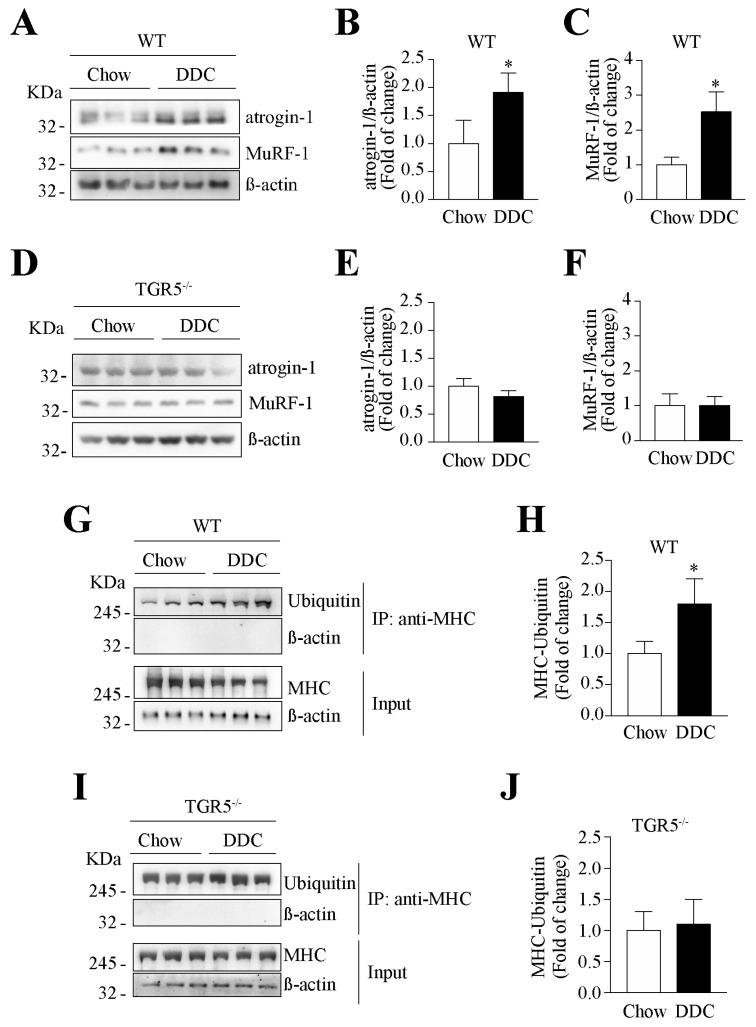
The increased expression and activity of the ubiquitin-proteasome system (UPS) in mice with CLD is dependent on TGR5 expression. C57BL/6J male WT and TGR5^−/−^ mice were fed with a chow or DDC-supplemented diet for 6 weeks. At the end of the experiments, TA muscles were excised and homogenized to evaluate the protein levels of E3 ubiquitin ligases atrogin-1 and MuRF-1 in WT (**A**) and TGR5^−/−^ (**D**) mice. β-actin was used as the loading control, and the molecular weight markers are depicted in kilodaltons (kDa). The quantitative analysis of atrogin-1 (**B**,**E**) and MuRF-1 (**C**,**F**) in WT (**B**,**C**) and TGR5^−/−^ (**E**,**F**) mice are shown. The results are expressed as fold of induction relative to the chow condition. The values represent the mean ± SEM (*n* = 7, * *p* < 0.05 vs. chow, *t*-test). UPS activity was determined by ubiquitinated-MHC protein levels, which were evaluated by immunoprecipitation with anti-MHC and Western blot with anti-ubiquitin in TA muscles from WT (**G**) and TGR5^−/−^ (**I**) mice. The upper panel shows the detection of ubiquitinated-MHC levels in the immunoprecipitated samples. The lower panel illustrates the immunodetection of MHC and β-actin in the input. The β-actin levels were used as the loading control, and the molecular weight markers are depicted in kilodaltons (kDa). The quantitative analysis of ubiquitinated-MHC in WT (**H**) and TGR5^−/−^ (**J**) is shown. The results are expressed as fold of induction relative to the chow condition. The values represent the mean ± SEM (*n* = 3, * *p* < 0.05 vs. chow, *t*-test).

We also evaluated the levels of UPS proteins atrogin-1 and MuRF-1 through Western blot analysis of TA muscles from WT (Figure 7A) and TGR5^−/−^ mice (Figure 7D). The quantitative analysis shows increased levels of atrogin-1 (Figure 7B, chow = 1.00 ± 0.24 vs. DDC = 1.94 ± 0.20) and MuRF-1 (Figure 7C, chow = 1.00 ± 0.13 vs. DDC = 2.52 ± 0.33) in the TA muscle from WT mice fed with the DDC diet. In the TA muscle from TGR5^−/−^ mice, there is no difference in atrogin-1 (Figure 7E, chow = 1.00 ± 0.08 vs. DDC = 0.81 ± 0.09) and MuRF-1 (Figure 7F, chow = 1.00 ± 0.19 vs. DDC = 1.01 ± 0.15) protein expression between the chow and DDC diets.

As MHC is a target protein for UPS during sarcopenia, we evaluated the effect of TGR5 expression on MHC ubiquitination through its immunoprecipitation. Figure 7G,I show the immunoprecipitation of ubiquitinated MHC in TA muscle samples from WT and TGR5^−/−^ mice, respectively. The quantification shows that the increased DDC-induced MHC immunoprecipitated in WT mice (Figure 7H, chow = 1.00 ± 0.02 vs. DDC = 1.88 ± 0.2) is abolished in TGR5^−/−^ mice (Figure 7J, chow = 1.00 ± 0.03 vs. DDC = 1.1 ± 0.3).

Together, the results show that the DDC diet increases the atrogin-1 and MuRF-1 protein levels and the ubiquitinated MHC levels in TA muscles from WT mice, whereas they are lost in TA muscles from TGR5^−/−^ mice.

### 2.7. The Muscular Oxidative Stress Induced by CLD Is Abolished in TGR5^−/−^ Mice

**Figure 8:**
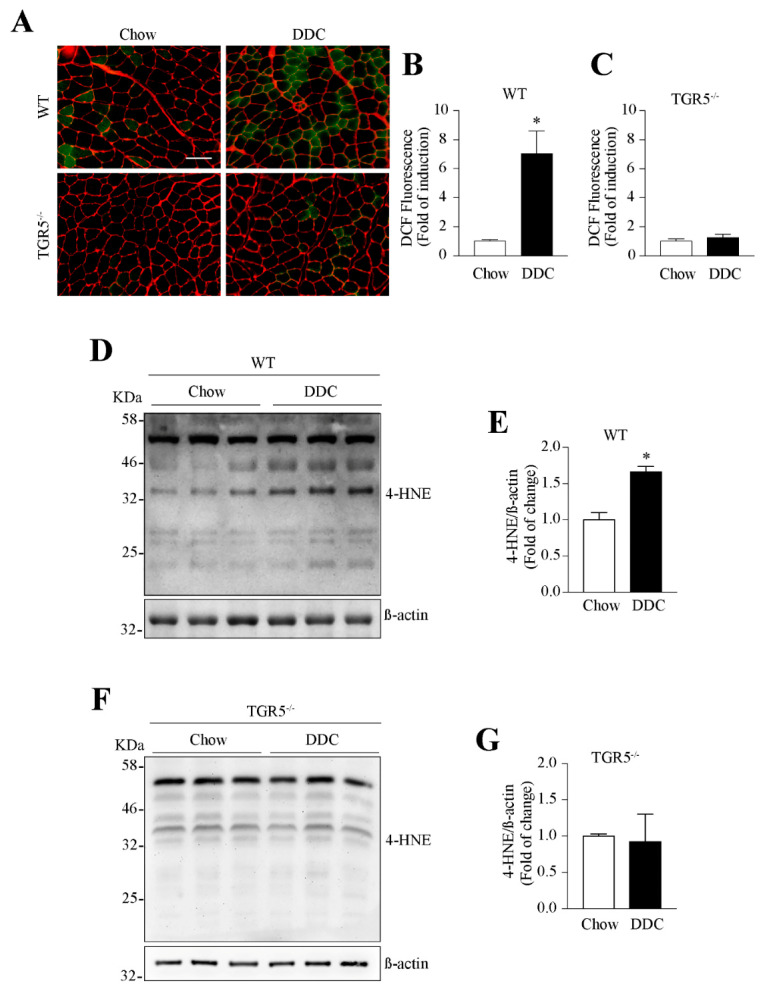
The muscular oxidative stress in mice with DDC-induced CLD is dependent on TGR5 expression. C57BL/6J male WT and TGR5^−/−^ mice were fed with a chow or DDC-supplemented diet for 6 weeks. TA muscle cryosections were incubated with the CM-DCF-H2 dye (green) to detect ROS. Incubation with WGA stain (red) was performed to delimit the sarcolemma (**A**). DCF-positive fibers were quantified and plotted for WT (**B**) and TGR5^−/−^ (**C**) mice. The scale bar corresponds to 100 µm. 4-HNE-modified protein levels were determined by Western blot in the TA muscle from WT (**D**) and TGR5^−/−^ (**F**) mice. β-actin was used as the loading control, and the molecular weight markers are depicted in kilodaltons (kDa). Quantitative analysis for 4-HNE detection in WT (**E**) and TGR5^−/−^ (**G**) mice. The results are expressed as fold of induction relatives to the chow condition. The values represent the mean ± SEM (*n* = 7, * *p* < 0.05 vs. chow, *t*-test).

We assessed the effect of TGR5 expression on the oxidative stress induced by the DDC diet. Figure 8A shows the ROS levels in the TA muscle from WT and TGR5^−/−^ mice. The quantitative analysis shows that the DDC-induced ROS increase observed in WT mice (Figure 8B, chow = 1.00 ± 0.07 vs. DDC = 7.04 ± 0.91) was abolished in TGR5^−/−^ mice (Figure 8C, chow = 1.00 ± 0.10 vs. DDC = 1.25 ± 0.14).

We also determined the effect of TGR5 expression on oxidative-dependent protein modifications by 4-HNE adducts. Figure 8D,F show the detection of 4-HNE-modified proteins by Western blot in WT and TGR5^−/−^ mice, respectively. The data quantification shows that the increase in 4-HNE reactivity induced by the DDC diet in WT mice (Figure 8E, chow = 1.00 ± 0.06 vs. DDC = 1.66 ± 0.04) is abolished in TGR5^−/−^ mice (Figure 8G, chow = 1.00 ± 0.02 vs. DDC = 0.92 ± 0.21).

These data suggest that TGR5 is necessary to generate the oxidative stress induced by the DDC diet in TA skeletal muscle.

## 3. Discussion

We have demonstrated in this study that TGR5 is a critical player in sarcopenia by DDC-induced CLD in mice. Using both in vivo and in vitro approaches, we have shown that all sarcopenia parameters, such as muscle strength and function, decreases in sarcomeric proteins, fiber diameter, and fiber transition are abolished in mice lacking TGR5 expression. Furthermore, TGR5 is essential to mediate oxidative stress and the increase in UPS levels and activity.

Our model of study corresponds to a CLD model caused by cholestasis (biliary duct obstruction). The antecedents in humans and mice show that one of the factors increased in cholestasis is the plasma BA [25,26]. One of the BA receptors is TGR5, which is expressed in the skeletal muscle. We show in this study that BA are increased in WT and TGR5^−/−^ mice fed with the DDC-diet. Furthermore, the results of muscle wasting and the TGR5 dependence in mice from this study are consistent with our previous report in which we demonstrated that BA induce muscle fiber atrophy via a TGR5-dependent mechanism [23]. Considering the fact that BA regulate muscle mass and that mice treated with the DDC diet have elevated plasma BA levels, we hypothesized that BA could be the soluble molecules upstream of TGR5 related to muscle wasting in this experimental model. Thus, TGR5 activation by BA would induce all features of sarcopenia in mice. This concept is supported by the current observation showing the absence of sarcopenia in mice lacking TGR5 expression. Previous studies indicate that other molecules could also participate in CLD-associated sarcopenia. Along this line, the hyperammonemia-myostatin axis has been described as one responsible for muscle wasting [27]. We did not evaluate this axis’s role in sarcopenia development in the DDC model in the present study. Thus, additional analysis could be performed in the future to clarify its involvement. However, a direct link has not been reported between hyperammonemia or myostatin and the TGR5 receptor.

Our data in this study, together with the previous report, suggest that the BA via TGR5 could be responsible for the muscle wasting caused in the DDC-induced sarcopenia model. We did not evaluate the activation of specific signaling pathways dependent on the BA/TGR5 axis in this work. However, we showed the increased activity of an electroporated-plasmid reporter responds to TGR5 activation in TA muscles from WT mice but not in TGR5^−/−^ mice (Supplementary Figure S2). Moreover, few antecedents of the mechanism downstream of TGR5 activation have been described. In brown adipose tissue, BA-dependent TGR5 activation induces the thyroid hormone activation by deiodinase 2 and generates an increment of energy expenditure by heat release [24]. The same study suggests that something similar could occur in skeletal muscle. However, there is not enough evidence to support this suggestion. There are antecedents in other tissues that indicate TGR5 can activate signaling pathways such as nuclear factor kappa B (NF-κB), protein kinase B (Akt), and extracellular signal-regulated kinase (ERK) [28]. Interestingly, these pathways are related to muscle mass regulation. Thus, we can speculate that in normal conditions, TGR5 activation regulates these pathways to maintain muscle mass, but it could induce atrophic effects when it is over-activated. However, additional studies are required to address this hypothesis.

TGR5 has different effects depending on the tissue in which it is expressed. Related to our study model and the consequences of TGR5 gene ablation on liver damage, it is well described that TGR5 is a hepatoprotective factor under pathological conditions. TGR5^−/−^ mice present an exacerbated immune-mediated cholestatic hepatic injury [29]. Accordingly to this hepatoprotective role of TGR5 when BA are increased, it was expected that the ablation of TGR5 would not improve cholestasis, which is consistent with our data concerning cholestasis, as shown in Table 1 (increased ALT, ALP, and total bilirubin) and Supplementary Figure S1 (increased plasma BA).

Regarding skeletal muscle, TGR5 is expressed in healthy skeletal muscle. Our data in this study indicate that TGR5 ablation protects the muscle from developing DDC-induced sarcopenia. To evaluate the specific role of muscular TGR5 itself, independent of TGR5 expression on other tissues such as the liver, studies in a muscle-specific TGR5-deficient mouse could be performed. Based on our previous reports, we can speculate that in a muscle-specific TGR5 deficient mouse, the DDC diet would induce the same degree of liver damage, cholestasis, and plasma BA levels than in wild-type mice, but with a protective effect on skeletal muscle, avoiding DDC-induced sarcopenia. Moreover, the evaluation of the TGR5 role in other models of sarcopenia independent of BA (such as sepsis, immobilization, or secondary to cardiac or renal failure) could indicate if muscular TGR5 ablation could have a protective role itself in muscle wasting.

A recent article described the role of TGR5 in the control of skeletal muscle function and the regulation of skeletal muscle cell differentiation [22]. Our group has corroborated the importance of TGR5 in skeletal muscle differentiation because when myoblasts were transfected with a short hairpin for TGR5, they remained undifferentiated (data not are shown). Despite this evidence, TGR5 is not essential for skeletal muscle formation in early development stages since adult TGR5^−/−^ mice are phenotypically normal. Therefore, it is possible to speculate that TGR5 could not be essential in the prenatal myogenesis process, in which the Pax3 transcriptional factor has a critical function [30], whereas TGR5 could have a crucial role in muscle differentiation associated with postnatal regeneration, in which Pax7 transcriptional factor seems to have an essential function [31]. Thus, further studies must be conducted to elucidate this possible differential function of TGR5.

It has recently been suggested that TGR5 could favor muscle hypertrophy, contrary to our findings in the present study [22]. Nevertheless, it is essential to note that our results were obtained from a model of CLD-induced sarcopenia, which is different from the healthy muscles presented in the other study [22]. It is plausible that in the case of CLD, the pathological increase in BA (in the DDC model, the BA concentration increased 1,200-fold compared with the control level) would aberrantly activate the TGR5 receptor. This fact is also observed for other receptors. Receptor for advanced glycation end-products (RAGE) signaling is critical in controlling muscle precursor activity during prenatal muscle development and muscle regeneration. However, the aberrant expression and/or activity of RAGE in adult skeletal muscle is associated with muscle wasting in response to aging, genetic disorders, inflammatory conditions, cancer, and metabolic alterations [32]. In the case of BA and TGR5 activation under pathological conditions, this event negatively affects muscle mass and strength, supported by the participation of BA/TGR5 in skeletal muscle atrophy [23]. It has been reported in the same line that in patients with non-alcoholic fatty liver disease, an inverse correlation between plasma BA levels and skeletal muscle volume has been observed [21].

The decrease in MHC protein levels correlates with higher UPS activity, evidenced by the increase in ubiquitinated MHC that leads to MHC degradation via the proteasome. The increased levels of atrogin-1 and MuRF-1 are typical muscle atrophy markers indicating UPS participation [33]. These events could be dependent on the oxidative stress present in sarcopenic muscle induced by the DDC diet. We have demonstrated the development of oxidative stress under the condition of DDC-induced sarcopenia, which is dependent on TGR5 expression. These results are consistent with previous studies indicating TGR5-dependent oxidative stress in different cell types [34,35,36]. We also previously reported the critical role of oxidative stress in DDC-induced sarcopenia, as evidenced by the prevention performed by the antioxidant treatment on muscle dysfunction and fiber alteration [14]. Oxidant sources and the effects on the antioxidant system in muscle under treatment with the DDC diet must be studied further. Along this line, BA via TGR5 induce NADPH oxidase (Nox) expression and activity, contributing to oxidative stress in skeletal muscle. However, the participation of other sources, such as mitochondria or xanthine oxidase, must also be analyzed.

We demonstrated the transition of type IIB fast-twitch fibers to type I slow-twitch fibers, passing through intermediate phenotypes, in TA muscles. However, the more oxidative fibers observed with DDC treatment (Figure 3E) are associated with fatigue resistance, which is opposite to our results in more fatigable muscle (Figure 4). Despite the apparent discrepancy between these results, there are several antecedents that can explain it:

(a) We determined fiber types by the detection of MHC isoforms. TA muscle is described as a fast muscle with predominant expression of the IIB isoform [37]. In our study, we detected that type IIB fast-twitch fibers were decreased by the DDC diet, whereas the slow type I fibers were increased. Although there was an increase in type I slow-twitch fibers (the fibers most resistant to fatigue), it is essential to consider that they only reach around 3%, relative to whole muscle fibers. Our data in this paper indicate that a higher increment occurs in fiber types IIA and IIXA (13.56% and 23.9%, respectively), which are more fatigable than fiber type I.

(b) The fatigue of muscle fibers is also dependent on their metabolism. The slower fibers that increased with DDC treatment in our study are associated with more oxidative metabolism, with high mitochondrial activity. However, data from our laboratory (not shown) indicate that the DDC diet induces mitochondrial dysfunction in TA muscles, directly affecting the oxidative metabolism of these fibers (manuscript in preparation). Among the effects observed are decreased ATP production, concomitantly with reduced levels of oxidative phosphorylation proteins (OXPHOS) in TA muscle of WT mice fed with DDC (but not in TGR5^−/−^ mice). Thus, the mitochondrial dysfunction and alterations in the fibers’ oxidative metabolism under DDC treatment could explain the fatigue observed on slow fibers despite the expression of MHC isoforms (I, IIA) associated with more oxidative metabolism.

(c) The fibers with oxidative metabolism, such as I and IIA, generate a high amount of ROS [38,39,40]. Furthermore, our data concerning mitochondrial dysfunction in TA muscles from DDC-treated WT mice indicate that it produces a higher amount of mitochondrial ROS. Thus, these facts can generate an imbalance between oxidants/antioxidants, inducing oxidative stress and resulting in a more fatigable phenotype [41,42,43,44,45]. Accordingly with these observations, our results in this paper (Figure 8) and our previous work [14] show that oxidative stress is directly involved in muscle dysfunction and atrophy in the DDC-induced sarcopenia model.

These events could explain the lessened muscle function (weightlifting, grip, and running tests) and the decreased force in isolated TA from WT mice fed with the DDC diet. Moreover, we demonstrated that these events are dependent on TGR5 expression. In several muscle-wasting conditions, such as cancer cachexia, diabetes, chronic diseases, and aging, type IIB fibers are more vulnerable to atrophy [46]. The transition of IIB fibers involves higher protein degradation with the participation of signaling transduction dependent on the forkhead box O (FOXO) family, autophagy inhibition, transforming growth factor-beta family, or NF-κB [47]. Further studies must be performed to explore the mechanism downstream of the TGR5 receptor involved in the fiber type shift in CLD.

Elucidating the role of TGR5 in CLD-related sarcopenia in our experimental model might have clinical implications. As previously mentioned, sarcopenia is a critical prognosis determinant in patients with advanced CLD, serving as an independent predictor of mortality and adverse outcomes both in the transplant waiting list and in the post-transplant setting [11,48]. Thus, strategies to ameliorate sarcopenia in patients with CLD may have a beneficial clinical impact. Current approaches include combining nutritional and physical interventions involving the guideline-based provision of appropriate energy and protein intake and engaging patients in moderate exercise programs. Some pharmacological interventions using testosterone, branched-chain amino acids, carnitine, or ammonia-lowering therapies have also been proposed, but more studies are needed. Based on the current findings, the use of TGR5 antagonism could be an additional potential strategy to limit muscle mass loss in patients with CLD, but this needs to be studied further. Of note, one TGR5 antagonist (SBI-115) has been successfully used in preclinical models of other liver diseases [49].

## 4. Materials and Methods

### 4.1. Animals

C57BL/6J WT male mice (16 weeks old) and C57BL/6J Gpbar1^−/−^ mice (referred to in this study as TGR5^−/−^ mice, 16 weeks old) were randomized and separated into experimental groups to perform three independent experiments. Mice were fed with a standard diet or a diet supplemented with 0.1% 5-diethoxycarbonyl-1,4-dihydrocollidine (DDC) (Sigma-Aldrich, St. Louis, MO, USA) for six weeks. The experimental conditions were two groups fed with a standard diet (chow) (WT chow and TGR5^−/−^ chow), and two groups fed with a supplemented diet with 0.1% DDC (WT DDC and TGR5^−/−^ DDC) [5,25]. Each experimental group contained five to nine mice. The muscles were obtained after dissection, and the samples were weighed, rapidly frozen in isopentane, and stored at −80 °C until processing. In some cases, the muscles were dissected and used for electrophysiological measurements. Our experiments strictly followed all international, national, and institutional suggestions and guidelines for animals’ care and use. Our studies and procedures with animals had the Animal Ethics Committee’s formal approval at the Universidad Andrés Bello (approval number 007/2016, March 2016).

### 4.2. Measurement of Fat, Lean Mass, and Body Water

The body composition of mice was measured through EchoMRI resonator analysis (Echo Medical Systems, Houston, TX, USA) at six weeks of treatment. The measurements allowed us to evaluate the percentage of fat, lean mass, and total and free water. The hydration index (HI) was calculated as follows: HI = (full water − free water)/lean.

### 4.3. Plasma Bile Acids Levels

Total plasma bile acids (BA) levels were using the colorimetric assay (Randox Laboratories Ltd., Kearneysville, WV, USA). Blood was drawn from the mouse’s tail (approximately 30 µL) and centrifuged at 1500× *g* for 10 min at 4 °C. Subsequently, the plasma was separated for the assay [50]. The absorption of the samples and the calibrator provided by the kit was measured at 405 nm at 60 and 120 s (A1 and A2 respectively), and the difference between the two values was calculated (ΔA = A2 − A1). The total bile acid concentration is calculated based on the following formula:

(ΔA_sample/_ΔA_gauge_) × [gauge] = [sample] µmol/L (1)

### 4.4. Parameters of Liver Injury

The colorimetric assay (Sigma-Aldrich, St. Louis, MI, USA) was used to measure serum alanine aminotransferase (ALT). Serum alkaline phosphatase (ALP) activity and total bilirubin levels were determined by routine clinical chemistry testing, as previously described [51].

### 4.5. Weightlifting Test

The mice were subjected to muscle strength measurement by means of a weightlifting test. The apparatus consisted of a series of chain links of increasing length attached to a ball of tangled fine wire. The number of links ranged from one to seven (15.5 up to 54.1 g). The mice grasped the different weights with their forepaws, and a score was assigned. The sum of all scores generated the final value. The average of three measures from each mouse was normalized by the bodyweight [14,52].

### 4.6. Running Test

The mice performed an aerobic exercise test. Animals ran on the treadmill tape (LE8610MTS, Panlab Harvard Apparatus, Barcelona, Spain) for 3 repeats of 5 min at 20 cm/s with a rest of 5 min. The test was recorded and videos were analyzed to calculate the residence time of mice in each of the two zones, which were defined as: zone distant from the starting point (high performance, HP) and the region closest to the starting point (low performance, LP) [5].

### 4.7. Rotarod

The mice performed an exercise trial on a rotarod (LE8205, Panlab Harvard Apparatus, Barcelona, Spain). To achieve the test, the mice were placed on the rotor with an initial rotation speed of 4 rpm. The fuel speed gradually increased to 35 rpm for 5 min. The total time that the animals stayed on the rod was measured [5].

### 4.8. Grip Strength Test

The grip strength test was used according to a protocol previously described to assess the maximum force on the animal’s back and front legs [53,54]. Following this protocol, 15 repetitions of force were performed on the front legs and 15 repetitions on the hind legs and the three best values were recorded in each experimental group.

### 4.9. Contractile Measurements in Isolated Skeletal Muscle

At the end of the treatment, mice were euthanized, and contractile properties of isolated skeletal muscle were evaluated by electrophysiology. Thus, the tibial anterior (TA) muscles were removed and maintained in an oxygenated Krebs–Ringer solution. Before the maximum tetanic force measurement, the maximum length for the highest contraction (Lo) and the micromanipulation of muscle length were determined. The muscles were stimulated with different frequencies between 1 and 150 Hz, 450 ms in duration, with 2 min of rest between each stimulus. The specific net force was calculated and normalized and expressed as mN/mm^2^ [14,55].

### 4.10. Western Blot

Thirty to eighty micrograms of proteins from TA muscles were used for Western blot analyses. The samples were separated by SDS-PAGE, transferred to PVDF membranes, and subjected to immunoblotting as previously described [56]. The primary antibodies used were mouse anti-MHC (1:1000 MF-20; Developmental Studies, Hybridoma Bank, University of Iowa, Iowa, IA, USA), mouse anti-troponin I (1:1000; Cell Signaling, Danvers, MA, USA), myosin type IIb (1:1000, BF-F3; Developmental Studies, Hybridoma Bank, University of Iowa, Iowa, IA, USA), rabbit anti-atrogin-1 (1:500, ECM Biosciences, Versailles, KY, USA), rabbit anti-MuRF-1 (1:500, ECM Biosciences, Versailles, KY, USA), goat anti-4-hydroxynonenal (4-HNE) (1:1000; Merck, Temecula, CA, USA), mouse anti-ubiquitin (1:5000, Santa Cruz, Dallas, TX, USA), and rabbit anti-β-actin (1:2000, Abcam, Cambridge, MA, USA). Furthermore, the membranes were incubated with the secondary antibody. The target protein’s reactivity was detected by means of a chemiluminescence procedure (Thermo Scientific, Waltham, MA, USA) through an image documentation system, Fotodyne (Fisher Scientific, St. Waltham, MA, USA). Quantification of the bands was performed by means of densitometric analysis using ImageJ software (NIH, Bethesda, MD, USA). The MF-20 hybridoma, a monoclonal antibody developed by Fischman, D.A., was obtained from the Developmental Studies Hybridoma Bank, created by the National Institute of Child Health and Human Development (NICHD) of the National Institute of Health (NIH) and maintained at The University of Iowa, Department of Biology, Iowa City, IA 52242, USA.

### 4.11. Co-Immunoprecipitation

The ubiquitinated MHC levels were determined. Briefly, total protein extracts from TA muscles were made with the non-denaturant lysis buffer and were immunoprecipitated with anti-MHC (1:100 MF-20; Developmental Studies, Hybridoma Bank, University of Iowa, Iowa, IA, USA). The antibody/antigen complex was separated from the sample using agarose beads coupled to protein G to isolate MHC. The immunoprecipitates were separated using SDS-PAGE and transferred to a PVDF membrane, which was subsequently incubated with antibodies against ubiquitin (1:5000, Santa Cruz, Dallas, TX, USA), MHC (1:1000 MF-20; Developmental Studies, Hybridoma Bank, University of Iowa, Iowa, IA, USA), and β-actin (1:2000, Abcam, Cambridge, MA, USA). Immunoreaction was visualized using chemiluminescence reagents (Thermo Scientific, Waltham, MA, USA), in Fotodyne FOTO/Analyst Luminary Workstation Systems (Fisher Scientific, St. Waltham, MA, USA). Densitometric analysis was determined using ImageJ software (NIH, Bethesda, MD, USA).

### 4.12. RT-qPCR

Total RNA was extracted from TA muscles using Chomczynski’s solution, and the purified RNA was transcribed into cDNA for standard methods, as we have previously described [56]. The cDNA obtained from reverse transcription was analyzed to evaluate the *TGR5* gene expression (forward: CAGGAGGCCATAAACTTCCA; reverse: GTCAGCTCCCTGTTCTTTGC) with SyBR Green using *18S* (forward: GTAACCCGTTGAACCCCATT; reverse: CCATCCAATCGGTAGTAGCG) as a housekeeping gene. PCR was performed in triplicate using an Eco Real-Time PCR System (Illumina, San Diego, CA, USA). The mRNA expression was calculated using the comparative ΔΔ Ct method [57].

### 4.13. Muscle Fiber Diameter Determination and Quantification

Cryosections (8 µm) of TA muscles were stained with wheat germ agglutinin (WGA) attached to Alexa-Fluor 594 (Thermo Fisher Scientific, Waltham, MA, USA) as previously described [14,58]. Images were acquired in a Motic BA310 fluorescence microscope (Motic, Hong Kong). The fiber size was determined by measuring Feret’s minimum diameter. This analysis was performed by manually selecting the fibers and using ImageJ software (NIH, Bethesda, MD, USA). The values were grouped in 5-µm diameter ranges.

### 4.14. Determination of Muscle Fiber Types

Immunofluorescence analysis of MHC expression was used to detect the fiber types in the TA muscles. Cross-sections (8 µm) were incubated with a mixture of primary antibodies against myosin type I (BA-F8; Developmental Studies, Hybridoma Bank, University of Iowa, Iowa, IA, USA), myosin type IIA (SC-71; Developmental Studies, Hybridoma Bank, University of Iowa, Iowa, IA, USA), and myosin type IIB (BF-F3; Developmental Studies, Hybridoma Bank, University of Iowa, Iowa, IA, USA) [5,59]. Afterwards, cross-sections were incubated with the mixtures of secondary antibodies to detect the specific isotypes of primary antibodies: Alexa Fluor 488 IgG1, Alexa Fluor 555 IgM, and Alexa Fluor 647 IgG2bI, which detect types IIA, IIB, and I fibers, respectively. Thereafter, rinses were performed, and finally immunoreactivity for the different types of fibers was captured in a Leica SP8 confocal microscope (Leica Microsystems Inc., Buffalo Grove, IL, USA). ImageJ software (NIH, Bethesda, MD, USA) was used to analyze images. The BA-F8, SC-71, and BF-F3 hybridoma monoclonal antibodies produced by Schiaffino, S. were obtained from the Developmental Studies Hybridoma Bank, created by the NICHD of the NIH and maintained at The University of Iowa, Department of Biology, Iowa City, IA, 52242. The fiber characterization and quantification were performed manually using ImageJ software (NIH, Bethesda, MD, USA). For the definition of fibers, the average fluorescence of unstained fibers (20 fibers) was determined and subtracted from each one of the channels to give the net fluorescence of each stain. The relative fluorescence of each fiber type was determined and expressed in arbitrary units (AU). Pure fibers were assigned a value of 1 (average of 20 fibers for each channel), whereas hybrid fibers were determined by fluorescence intensity relative to the pure fiber.

A protocol of manual quantification was used as previously described [60]. The image composed of the three channels was analyzed and fibers were scored as positive/negative in each of the three channels and defined as IIA, IIB, or I. In comparison, fibers that were scored as negative under all three channels were classified as type IIX. In the case of hybrid fibers, they were defined as follows:

- Positive fibers on both Alexa Fluor 488 (IIA) and 647 (I) were classified as I/IIA hybrid fibers.

- Positive fibers on Alexa Fluor 488 (IIA), but with less fluorescence intensity relative to the pure fiber respective, were classified as type IIA/X hybrid fibers.

- Positive fibers on Alexa Fluor 555 (IIB), but with less fluorescence intensity relative to the pure fiber, were classified as type IIB/X hybrid fibers.

For the quantification, the percentage of each fiber type was calculated considering the number of specific fibers to the total number of fibers (with the sum of all fibers types corresponding to 100%).

### 4.15. Skeletal Muscle Electroporation and Luciferase Activity

TA muscles from WT y TGR5^−/−^ mice were electroporated with pGL4.29, a luciferase plasmid reporter containing CRE binding sites (Promega, Madison, WI, USA), together with pRL-SV40 (Promega, Madison, WI, USA). This last plasmid was used to normalize luciferase activity. Briefly, the mice fed with a chow or DDC-supplemented diet for six weeks were anesthetized in a chamber with isoflurane and kept with the mask on during the entire procedure. The hindlimbs were shaved, and 15 µL of hyaluronidase (2 mg/mL) was injected intramuscularly in TA. Then, mice were maintained in a cage for their recovery. After 1 h, the mice were anesthetized again, and 30 µg of pGL4.29 plus 15 µg of pRL-SV40 were injected into the TA muscle. Conductive gel was applied around the TA muscle, and Tweezertrodes electrodes (BTX Molecular Delivery Systems, Holliston, MA, USA) were put over this zone. The muscle was stimulated with 8 pulses of 200 V/cm for 20 ms and 1 Hz using an ECM 830 Square wave electroporation system (BTX Molecular Delivery Systems, Holliston, MA, USA). The mice were recovered, and after ten days, TA muscle was dissected and homogenized using the passive lysis buffer of a dual-luciferase reporter assay system (Promega, Madison, WI, USA). The luciferase activities were registered following the supplier’s instructions using a Glomax luminometer (Promega, Madison, WI, USA) [61].

### 4.16. Statistical Analysis

The statistical analysis of the data was performed with Prism 8.0 analysis software (GraphPad Software, San Diego, CA, USA). The normality of the data was determined. Normal data were analyzed with a *t*-test to compare two groups, and to analyze two or more groups, one or two-way ANOVA was used as appropriate, with a Tukey or Sidak post-hoc test. Differences were considered significant when the *p*-value was <0.05.

## 5. Conclusions

In conclusion, our results show decreased muscle mass and strength in mice with DDC-induced CLD, events that are dependent on the expression of the membrane BA receptor TGR5. In this study, we focused on determining TGR5 deficiency in muscular wasting, and we have not conducted any experiments with liver tissue to evaluate the effects of TGR5 ablation.

Our findings suggest that among the underlying mechanisms that can explain muscle weakness in our model are the decreased sarcomeric proteins, oxidative stress, and the fast-to-slow shift in muscle fiber type composition. Additional mechanisms at play in determining muscle dysfunction in CLD may be involved but were not explored in our study. Among these could be alterations in motoneuron activity due to peripheral neuropathy derived from liver disease or metabolic changes that affect mitochondrial function in skeletal muscle [62,63]. These factors could be explored in subsequent studies.

Thus, sarcopenia induced by a DDC-supplemented diet in mice is dependent on TGR5 receptor expression.

## Figures and Tables

**Table 1:** Physiological parameters in mice with DDC-induced chronic liver disease (CLD).

Physiological Parameters		*Wild-Type* (WT)		TGR5^−/−^	
		Chow	DDC	Chow	DDC
Muscle mass (mg)	TA	30.4 ± 2.3	23.2 ± 2.9 *	28.4 ± 3.9	29.4 ± 2.0
	GAST	86.0 ± 9.2	62.7 ± 16.2 *	93.0 ± 4.2	89.0 ± 2.2
	EDL	9.9 ± 1.6	7.8 ± 1.1 *	8.9 ± 1.9	8.3 ± 1.0
	SOL	8.5 ± 1.7	6.2 ± 1.0 *	7.5 ± 1.0	8.5 ± 1.1
Liver mass (g)		1.3 ± 0.1	1.9 ± 0.2 *	1.1 ± 0.2	2.2 ± 0.3 ^#,^*
ALT (IU/L)		167 ± 16	545 ± 32 *	145 ± 18	497 ± 29 ^#,^*
ALP (IU/L)		90 ± 10	982 ± 74 *	119 ± 22	1091 ± 98 ^#,^*
Bilirubin total (mg/dL)		0.3 ± 0.1	1.8 ± 0.3 *	0.5 ± 0.2	2.3 ± 0.4 ^#,^*
Fat (g)		1.31 ± 0.09	0.85 ± 0.03 *	1.86 ± 0.18 *	1.79 ± 0.13 *
Lean (g)		12.9 ± 0.3	9.3 ± 0.3 *	11.3 ± 0.7	10.7 ± 0.3
Water (g)	Total	11.2 ± 0.3	8.0 ± 0.3 *	9.3 ± 0.3	8.9 ± 0.3 *
	Free	0.107 ± 0.061	0.075 ± 0.053	0.073 ± 0.025	0.095 ± 0.051
Hydration ratio (%)		85.9	85.2	81.7	82.3
Glycemic index (g/dL)		143.0 ± 5.6	78.8 ± 4.4 *	162.4 ± 8.4 *	81.0 ± 4.6 ^#,^*

The values correspond to mean ± SEM (*n* = 7–9, * *p* < 0.05 relatives to WT-chow, # *p* < 0.05 relative to TGR5^−/−^-chow. Two-way ANOVA, Tukey’s multiple comparison test.

## Supplementary Materials

The following are available online at https://www.mdpi.com/1422-0067/21/21/7922/s1.
