# Supplementary material for: Sarcopenia Induced by Chronic Liver Disease in Mice Requires the Expression of the Bile Acids Membrane Receptor TGR5
Source: Int J Mol Sci. 2020 Oct 25;21(21):7922. doi: 10.3390/ijms21217922 (PMC7662491; doi:10.3390/ijms21217922)
Supplement: Supplementary file 1 [file ijms-21-07922-s001.pdf]

International Journal of *Molecular Sciences*

MDPI

Article

Sarcopenia Induced by Chronic Liver Disease in Mice Requires the Expression of the Bile Acid Membrane Receptor TGR5

Johanna Abrigo[1,2,3], Fabián Campos[1,2,3], Francisco Gonzalez[1,2,3], Francisco Aguirre[1,2,3], Andrea Gonzalez[1,2,3], Camila Huerta-Salgado[1,2,3], Sabrina Conejeros[1,2,3], , Felipe Simon[2,4,5], Marco Arrese[6], Daniel Cabrera[6,7], Alvaro A. Elorza[2,8] and Claudio Cabello-Verrugio[1,2,3*]

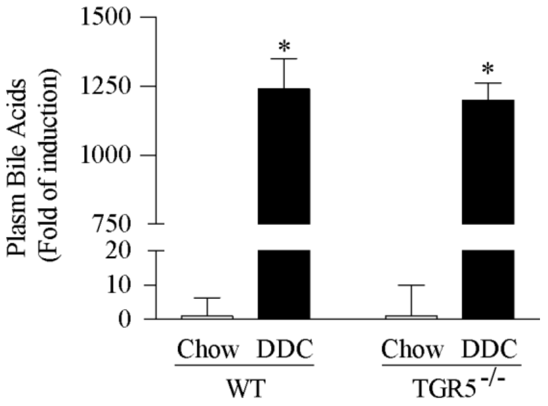

Figure S1. The levels of plasma bile acids (BAs) are increased in mice with DDC-induced CLD. C57BL/6J male mice WT and TGR5-/- were fed with a chow or DDC-supplemented diet for 6 weeks. When the experiments were completed, the amount of total BAs in plasma was measured. The results are expressed as fold of induction relative to the chow condition. The values represent the mean ± SEM (n = 7-9, *p < 0.05 vs. chow, t-test).

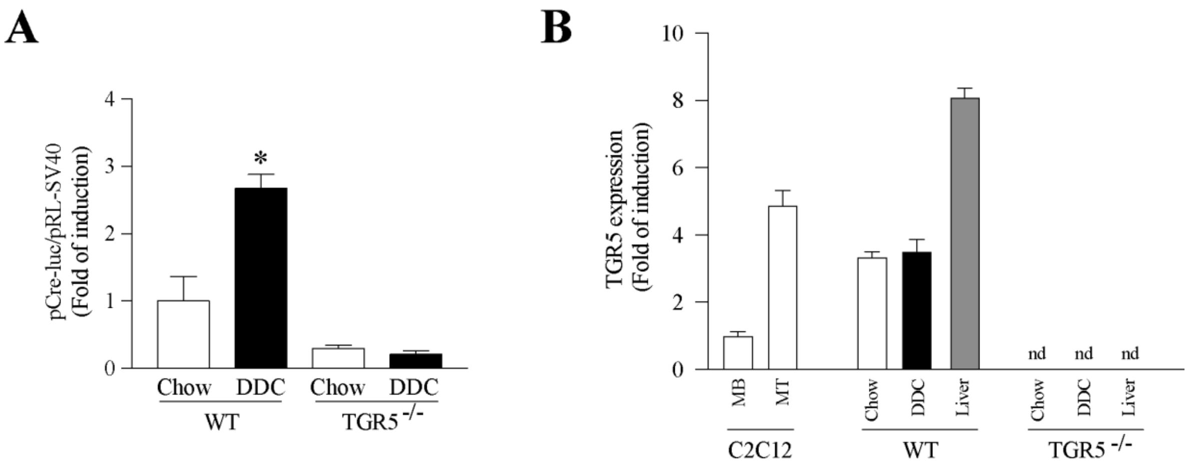

Figure S2. TGR5 activation in muscle from mice with DDC-induced CLD. C57BL/6J male mice WT and TGR5-/- were fed with a chow or DDC-supplemented diet for 6 weeks. (A) At 6 weeks, TA muscles were electroporated with plasmid reporter pCRE-luc and pRL-SV40. Luciferase activity was measured from TA extracts and normalized by luciferase activity from renilla (pRL-SV40). The results are expressed as fold of induction relative to the WT-chow condition. The values represent the mean ± SEM (n = 3, *p < 0.05 vs. chow, #P < 0.05 vs. WT DDC, Two-way ANOVA, Tukey's multiple comparison test). (B) mRNA expression of the TGR5 receptor was evaluated by RT-qPCR using the 18S gene as housekeeping gene. cDNA from extracts of liver and C2C12 myoblasts (MB) and myotubes (MT) were used as positive controls. The values are shown as a fold of change relative to MB and expressed as the mean ± SEM (n = 7-9, * p < 0.05 relatives to MB, two-way ANOVA, Tukey's multiple comparison test).
